# Labial synechiae in geriatric patients with turner syndrome: A case report

**DOI:** 10.1016/j.ijscr.2020.03.010

**Published:** 2020-03-07

**Authors:** Johannes Cansius Prihadi, Yuvi Wahyudi

**Affiliations:** a Department of Surgery, Division Urology, Faculty of Medicine, Atmajaya Catholic University, Jakarta, Indonesia; b Department of Urology, St. Carolus Hospital, Jakarta, Indonesia

**Keywords:** Labial synechieae, Turner syndrome, Females, Geriatry, Vaginal reconstruction, Case reports

## Abstract

**Introduction:**

Turner syndrome (TS) is a chromosomal condition that affects development in females, resulted from a complete or partial absence of the second X chromosome or from structural anomalies (mosaicism) of one X chromosome. TS is one of the most common sex chromosome disorders among live-born female births.

**Case presentation:**

We presented with a 63 y.o. Female, who admitted to our Hospital with a chief complaint of pain when urinating and frequent haematuria. She has a history of Turner Syndrome (45 XO). Local examination in the lithotomy position revealed labial agglutination involving the labia majora. We did a Cystoscopy to evaluate the bladder and urethra, and then biopsied the tissues. Vaginal Reconstruction was performed to repair the Labial Synechiae. The biopsy showed that this histological finding is consistent with Follicular Cystitis that could cause by chronic UTIs.

**Discussion:**

Labial Synechiae (LS), or Labial agglutination is a disorder of the female genitalia characterized by thin, membranous adherence of the labia. LS usually noted in female with numerous UTIs and vaginal infections. Goel et al confirmed that LS is not a congenital disorder. In a retrospective study, they found out that LS is probably caused by multiple numerous UTIs that may cause a membrane to formed around the labia. The negligence and chronicity of this condition may have thickened the synechiae, making it persist for a long time, and can actually cause a symptom to appear.

**Conclusion:**

There is no literature to suggest any link between TS and LS directly.

## Highlights

• Turner Syndrome is a chromosomal condition that affects development in females, occurring in approximately 1/2500 live female births.
• Labial Synechiae usually noted in female with numerous UTIs and vaginal infections.
• Synechiae is probably the result of multiple recurrent infections that could cause a chronic inflammation, denudating epithelial cells forming an agglutination of both labia.
• There is no literature to suggest any link between TS and LS directly.

## 1 Introduction

Turner syndrome (TS) is a chromosomal condition that affects development in females. It is caused by the haplo-insufficiency of some or all genes on the X chromosomes. This status results from a complete or partial absence of the second X chromosome or from structural anomalies (*mosaicism*) of one X chromosome [1,2]. TS is one of the most common sex chromosome disorders and occurs in approximately 1/2500 live-born female births. The most characteristic clinical features of TS are short stature and gonadal dysgenesis, but many organ systems and tissues such as cardiovascular, endocrine, gastrointestinal, renal, neurological, psychiatric, dermatologic, and orthopedic and muscular systems are also affected. Most girls and women with Turner syndrome have normal intelligence. Developmental delays, nonverbal learning disabilities, and behavioral problems are possible, although these characteristics vary among affected individuals [2]. A small percentage of females with Turner syndrome retain normal ovarian function through young adulthood, and most women essentially have normal external and internal vagina. Girls with Turner syndrome may have some malformation of the kidneys [3]. Although these abnormalities generally don't cause medical problems, they may increase the risk of high blood pressure and recurrent urinary tract infections. Multiple recurrent infections could cause a chronic inflammation. Synechiae is probably the result of vulvovaginitis or chronic dampness resulting from urinary incontinence [4,5]. Few layers of epithelial cells may denudate from the labia minora and apposition of the eroded areas can result in labial synechiae formation [6]. The relative hypoestrogenic state has been postulated to be the cause behind its prevalence in childhood and in elderly women [[5], [6], [7]].

Labial synechiae (LS), also referred to as labial adhesion or labial agglutination, is a disorder of the female genitalia characterized by thin, membranous adherence of the labia [6,8]. Typically, the fusion originates from the posterior fourchette and advances toward the clitoris. Complete labial fusion may conceal the vaginal introitus completely. Partial labial fusion is also possible and may occur near the posterior fourchette [6]. Labial adhesions can form, leading to urinary problems, and fissures and skin splitting often lead to sexual difficulties and superficial dyspareunia. LS often remains underdiagnosed, usually because the patient shies away from medical attention, but also as the condition may not be readily identified by general physicians or endocrinologists [1]. Genital skin, including skin over the mons pubis and perineum is often involved, giving a typical figure of eight or hourglass pattern. Skin changes occur only externally and the vagina is not affected. When LS remains untreated, labia minora gradually atrophy, labia majora become flat, adhesions or strictures can form at the introitus or around the prepuce, leading to a hooded clitoris [1,6]. In this Case Report, our work has been reported in line with SCARE Criteria [9].

## 2 Case report

A 63 y.o. Female, admitted to our Hospital with a chief complaint of pain when urinating. There were no symptoms of urinary obstruction, or post micturition dribbling of urine. But, she has complaints about traces of blood appearing in her urine in multiple occasions (frequent hematuria). She has a long history of recurrent Urinary Tract Infection (UTI) since she was a girl. Her medical history revealed that the patient had been diagnosed with a genetic abnormality called Turner Syndrome (45 XO). On physical examination, she has a short stature and a low hairline at the back of her neck. According to the patient, aside from recurrent and multiple UTIs, she never had any major complaint about her *genitourinary* tract, as well as any other abnormalities.

Further examination of her genitals revealed a labial synechiae/agglutination involving the labia majora. No signs of any inflammation or infection on the external area. Genitourinary Ultrasound also revealed no abnormal findings. We then decided to perform a Cystoscopy and biopsy the bladder wall to obtain more data.

## 3 Operation report

**Fig. 1:**
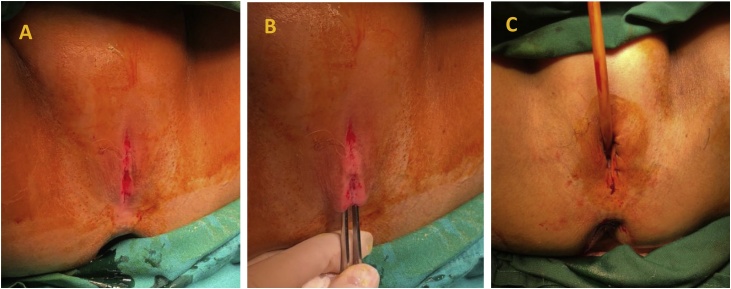
Findings during Vaginal Reconstruction Surgery of patient with Labial Synechiae, with a history of Turner Syndrome. (A) The outer appearance of patient’s vagina before reconstruction was made. (B) Vaginal Canal was identified and the fused labia were excised. (C) Urethral External Orifice was identified, followed by insertion of 22 Fr Catheter.

The Operation was performed under Spinal Anesthesia. Externally, the vagina looks abnormal, with two labia majora fused (Fig. 1-A). We start by excising the fused labia (B), exposing access into patient’s urethra, and then Cystoscopy was performed. We found a hyperemic bladder throughout the bladder wall and then we did a biopsy. After that, we did a vaginal reconstruction repair to fix the fused labia and urethral catheter was inserted (C).

**Fig. 2:**
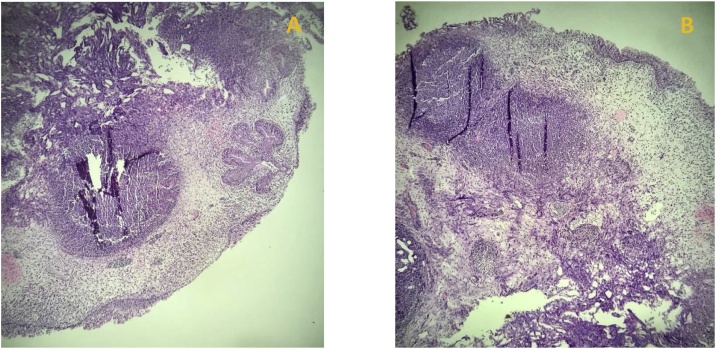
Histopathological analysis of the patient’s bladder biopsy. (A) Specimen consists of polypoid mucosal tissue. The surface is coated with partially-eroded urothelial cells. (B) Lamina Propria swells with sudden and chronic inflammatory cells, with some lymphoid follicles containing Germinal Center. Von Brunn nests were identified. Vessels appear congested.

The biopsy result came back a week after surgery. The vesical wall biopsy consists of polypoid-shape mucosal tissue, with some urothelial epithelium that looks eroded. Lamina Propria was filled with acute inflammation cells, with some germinal center within the lymphoid follicle. *Von Brunn nests* were identified. The vessels around looked congested. This histological finding is consistent with Follicular Cystitis that could caused by chronic UTIs. The biopsy tissues are shown in Fig. 2(A & B).

## 4 Discussion

Turner Syndrome (TS) is one of the most common chromosomal disorders in humans, especially female. It is caused by complete deletion of X chromosome, or *mosaicism* of the short arm of the X chromosome. TS is commonly diagnosed at birth or childhood due to some apparent anomalies. However, some will get to be diagnosed later due to delayed puberty and amenorrhea [1,2]. Labial Synechiae (LS), or Labial agglutination is a disorder of the female genitalia characterized by thin, membranous adherence of the labia [6,8]. LS usually noted in female with numerous UTIs and vaginal infections. Goel et al. confirmed that LS is not a congenital disorder. In a retrospective studies, they found out that LS is probably caused by multiple numerous UTIs that may cause a membrane to formed around the labia [6]. The negligence and chronicity of this condition may have thickens the synechiae, making it persist for a long time, and can actually cause a symptom to appear [[4], [5], [6], [7]]. There are no literatures to suggest any link between TS and LS directly. In many studies, patients with TS who has reached adulthood, usually has normal renal function and presented as normal lab result [1]. There was no relationship with whether any accompanying renal malformation was present. Abnormality found in internal genitalia and the urinary system is not associated with LS [1,2,6].

In this report, based on the findings from patient’s vesical biopsy, we thought that patient’s history of recurrent numerous UTIs contribute greatly in developing her LS condition. Patient’s diagnosis of LS was found at adulthood, since the patient herself rarely seeks any medical attention, making this a delayed diagnosis to establish.

We believe that all TS patients especially with urinary problems should require a continuous follow-up, with special attention and specific education related to this rare genetic condition to ensure their quality of life and well being, especially for patients who shy away from any medical help [1,2]. Research and studies should be continued on urinary and renal problems in TS patients, since they are relatively limited right now [2].

## Declaration of Competing Interest

We don’t have any conflicts of interest.

## Sources of funding

We fund the research all by ourselves.

## Ethical approval

We hereby state that we have the approval from our Hospital Ethical Committee and the patient herself.

## Consent

Written informed consent was obtained from the patient for publication of this case report and accompanying images.

## Author’s contribution

1. Study Conception and Design: Prihadi, Wahyudi
2. Acquisition of Data: Prihadi, Wahyudi
3. Analysis and Interpretation of Data: Prihadi, Wahyudi
4. Drafting of Manuscript: Prihadi, Wahyudi
5. Critical Revision: Prihadi, Wahyudi

## Registration of research studies

Our Case Report did not involve any human trials or studies.

## Guarantor

Johannes Cansius Prihadi, MD, PhD.

## Provenance and peer review

Not commissioned, externally peer-reviewed.
